# Hypoxia inducible-factor 1 alpha regulates neutrophil recruitment during fungal-elicited granulomatous inflammation

**DOI:** 10.3389/fcimb.2022.1005839

**Published:** 2022-10-06

**Authors:** Sara da Silva-Ferreira, Cláudio Duarte-Oliveira, Daniela Antunes, Catarina Barbosa-Matos, Ana Mendes-Frias, Egídio Torrado, Sandra Costa, Ricardo Silvestre, Cristina Cunha, Agostinho Carvalho

**Affiliations:** ^1^ Life and Health Sciences Research Institute (ICVS), School of Medicine, University of Minho, Braga, Portugal; ^2^ ICVS/3B’s– PT Government Associate Laboratory, Guimarães/Braga, Portugal

**Keywords:** hypoxia inducible-factor 1 alpha, *Aspergillus*, granuloma, neutrophils, chronic fungal disease

## Abstract

Chronic pulmonary aspergillosis (CPA) is a devastating disease with increasing prevalence worldwide. The characteristic granulomatous-like inflammation poses as the major setback to effective antifungal therapies by limiting drug access to fungi. These inflammatory lung structures are reported to be severely hypoxic; nevertheless, the underlying mechanisms whereby these processes contribute to fungal persistence remain largely unknown. Hypoxia-inducible factor 1 alpha (HIF-1α), besides being the major cellular response regulator to hypoxia, is a known central immune modulator. Here, we used a model of *Aspergillus fumigatus* airway infection in myeloid-restricted HIF-1α knock-out (*mHif1α^-/-^*) mice to replicate the complex structures resembling fungal granulomas and evaluate the contribution of HIF-1α to antifungal immunity and disease development. We found that fungal-elicited granulomas in *mHif1α^-/-^* mice had significantly smaller areas, along with extensive hyphal growth and increased lung fungal burden. This phenotype was associated with defective neutrophil recruitment and an increased neutrophil death, therefore highlighting a central role for HIF-1α-mediated regulation of neutrophil function in the pathogenesis of chronic fungal infection. These results hold the promise of an improved capacity to manage the progression of chronic fungal disease and open new avenues for additional therapeutic targets and niches of intervention.

## Introduction

Aspergillosis defines a spectrum of fungal infections usually caused by the ubiquitous fungus *Aspergillus fumigatus* and that is often associated with high mortality rates in immunocompromised patients (Arastehfar et al., 2021). Chronic pulmonary aspergillosis (CPA) is one form of aspergillosis, thought to affect about 3 million people worldwide (Hayes and Novak-Frazer, 2016). CPA usually occurs in patients with an underlying lung disease and is characterized by a chronic progressive fungal infection (Denning et al., 2016). In its most common phase, CPA takes the form of fungal balls within the lungs, termed aspergilloma or cavitary aspergillosis (Hayes and Novak-Frazer, 2016). This disease might progress further and originate granulomatous structures within lung cavities, surrounded by fibrotic areas and necrotic tissue (Denning et al., 2003). In recent years, increasing resistance to antifungal therapy further intensified the demand for novel therapies and biomarkers dictating susceptibility, and complementary methods to diagnose CPA. However, despite recent efforts, the underlying disease mechanisms in CPA remain a largely unknown topic.

Hypoxia-inducible factor 1 alpha (HIF-1α) is a transcription factor extensively studied for its broad regulatory roles both in cell metabolism and response to hypoxic conditions. Under normal conditions, HIF-1α is hydroxylated by prolyl-4-hydroxylases (PHDs) and marked for degradation by the Von Hippel–Lindau E3 ubiquitin ligase complex (Jaakkola et al., 2001). However, when oxygen levels drop, PHDs are inhibited and HIF-1α translocates to the nucleus, where it dimerizes with HIF-1β, inducing target gene expression (Greer et al., 2012). HIF-1α can also be stabilized by oxygen-independent mechanisms, such as lipopolysaccharide signalling through Toll-like receptor 4 (Blouin et al., 2004), succinate accumulation (Koivunen et al., 2007), iron depletion (Hartmann et al., 2008) and increased levels of mitochondrial reactive oxygen species (Movafagh et al., 2015).

HIF-1α is a pivotal immuno-metabolic regulator, since it orchestrates immune cell activation towards an inflammatory state by promoting glycolysis and pro-inflammatory cytokine secretion, along with enhanced phagocytosis and microbicidal activity (Cheng et al., 2014). HIF-1α is also known to portray a significant effect on granule proteases and release of antimicrobial peptides, as well as on nitric oxide production through nitric oxide synthase upregulation (Peyssonnaux et al., 2005). In neutrophils, HIF-1α was shown to promote the formation of extracellular traps (McInturff et al., 2012) and to induce survival and resistance to apoptosis (Walmsley et al., 2005). In the context of *A. fumigatus* infection, a few studies evidenced that this transcription factor is a key player in innate immunity by influencing cytokine secretion, namely tumour necrosis factor (TNF), interleukin (IL)-1β and IL-6 by dendritic cells (Fliesser et al., 2015), as well as neutrophil recruitment to the lungs through the induction of C-X-C motif chemokine ligand 1 (CXCL1) (Shepardson et al., 2014). More recently, the fungal melanin-driven upregulation of glucose metabolism, which is required for optimal fungal clearance, was found to depend on HIF-1α activity (Goncalves et al., 2020). Notwithstanding, the exact role of HIF-1α in the onset and progression of CPA remains unclear.

Here, we hypothesized that HIF-1α might regulate the susceptibility to chronic fungal disease and influence the development of *A. fumigatus* airway infection. By resorting to a mouse model of chronic fungal stimulation using agar beads containing *Aspergillus* conidia (*Af*-beads) (Urb et al., 2015), we demonstrated that HIF-1α influences the formation of granulomas and plays a role both in fungal clearance and in the control of hyphal growth.

## Methods

### Mice

All *in vivo* experiments were performed on eight to twelve-weeks old C57BL/6 and HIF-1α myeloid-restricted knock-out (*Hif1a* ^fl/fl^-LysMcre^+/+^) mice. Animals were housed and bred at the ICVS Animal Facility under specific pathogen-free conditions, fed *ad libitum* and kept under light/dark cycles of 12 h, temperature of 18–25 °C and humidity of 40–60%. All animal experimentation was performed following biosafety level 2 protocols approved by the Institutional Animal Care and Use Committee of University of Minho with ethical and regulatory approvals consented by SECVS (no. 074/016). Furthermore, all experiments followed the EU-adopted regulations (Directive 2010/63/EU) and were conducted according to the guidelines sanctioned by the Direção-Geral de Alimentação e Veterinária (DGAV).

### Mouse model of *Aspergillus fumigatus* airway infection

To mimic fungal-elicited granulomatous structures, infection was performed with agar beads containing *A. fumigatus* CEA17 conidia, as previously described (Urb et al., 2015). Briefly, sex-matched mice were anesthetized with 75 mg/kg of ketamine and 1 mg/kg of medetomidine. Then, 50 μL of a bead suspension either containing 2.5×10^6^ conidia or the equivalent volume of naked beads (beads without conidia) were administered intratracheally. At indicated timepoints, mice were anesthetized and perfused with PBS. The left lung was fixated in formalin 10% (Sigma-Aldrich) for 48 hours at room temperature, processed and embedded in paraffin for histological analysis. The accessory lobe was frozen immediately and used for RNA extraction.

### Histological analysis of murine granulomas

Lung sections were stained with haematoxylin and eosin (H&E) to assess inflammation and granuloma area. The areas of the granulomas were measured by subtracting the area of the beads to the overall area of granulomatous structures. Areas were measured resorting to Image Processing and Analysis in Java (ImageJ) software, version 1.52p. The histological sections as well as the H&E staining were performed by the Histological Unit of ICVS.

### Fungal burden quantification

To assess fungal burden in the lungs, chitin was quantified as described (Lehmann and White, 1975). Briefly, the right lung lobes were removed, weighed, and homogenized in 1 mL of sterile PBS with a tissue homogenizer (Glas-Col). The resulting pellets were re-suspended in 4 mL of 3% sodium dodecyl sulphate and incubated at 100°C for 15 min. After a wash with distilled water, the pellets were re-suspended with 3 mL of KOH 21.4 M and incubated at 130°C for 1h. Then, 8 mL of ice-cold 70% ethanol were added and shaken to form a single phase. After 15 min on ice, 0.3 mL of Celite suspension (Sigma) were added and left to stand for 2 min. Following a wash with ice-cold 40% ethanol, and two washes with distilled water, the pellets were re-suspended in 0.5 mL distilled water. A standard of 10 µg/mL glucosamine (Sigma) was prepared and 0.5 mL of 5% NaNO_2_ and 0.5 mL of 5% KHSO_4_ were added to each sample. After 15 min of incubation with agitation, 0.6 mL of supernatant from each sample were mixed with 0.2 mL of 12.5% ammonium sulfamate for 5 min. Next, 0.2 mL methylbenzothiazolone (MBTH) (50 mg in 10 mL distilled water) were added to each sample followed by a 3 min incubation at 100°C, before 0.2 mL of 0.83% FeCl_3_◦6H_2_O were added to each sample. The optical densities (OD) were read at 650 nm after 25 min. Data were expressed as µg glucosamine according to the formula: x=[5(A-B)]/(G-W), where A is OD_650_ of the samples from infected animals, B is the mean OD_650_ of the samples from uninfected animals, G is OD_650_ of the glucosamine standard, and W is OD_650_ of the water standard. To further assess conidia germination and hyphal growth within the lungs of mice, tissue sections were stained with Periodic acid-Schiff (PAS). The histological sections, as well as the PAS staining were performed by the Histological Unit of ICVS.

### Quantification of cytokines and chemokines

Lung homogenates were used to quantify TNF, IL-6 and IL-1β by Enzyme-Linked Immunosorbent Assay (ELISA) using ELISA MAX Deluxe Set kits (BioLegend), according to the manufacturer’s instructions. Chemokines were also quantified in lung homogenates using the LEGENDplex Mouse Proinflammatory Chemokine Panel (BioLegend), according to the manufacturer’s instructions. Samples were acquired on a BD LSRII using FACS Diva software (Becton and Dickinson). Data analysis was performed using LEGENDplex Data Analysis Software (version 8, BioLegend).

### Leukocyte recruitment

To analyse lung-infiltrating leukocytes in the lungs, the right lung lobes of mice inoculated with *Af*-beads were collected and minced into small fragments with scalpels. The resulting lung fragments were enzymatically digested with 1 mg/mL collagenase D (Sigma-Aldrich) and mechanically disrupted by multiple passages through a 70 μm strainer (Corning Inc.). Contaminating erythrocytes were lysed and leukocytes isolated by Percoll (GE Healthcare Bio-Sciences Ab) density gradient. Cells were incubated on ice with Zombie Violet viability dye (BioLegend) for 30 min to assess viability. For surface staining, cells were incubated on ice for 30 min with fluorochrome-labelled monoclonal antibodies indicated below. After fixation with 1% paraformaldehyde solution for 5 min, cells were re-suspended in FACS buffer (PBS, 2% FBS, 2 mM ethylenediaminetetraacetic acid). Myeloid subpopulations in the lung were identified using a combination of the following antibodies: BV510 anti-mouse CD45 (clone 30-F11), PE-Cy7 anti-mouse CD11b (clone M1/70), BV711 anti-mouse Ly-6G (clone 1A8) (all from BioLegend), PE anti-mouse Siglec-F (clone E50-2440) (BD-Pharmingen). Data were acquired with a BD FACS LSRII instrument (Becton Dickinson) and analysed with FlowJo software (Tree Star Inc).

### RNA isolation and quantitative RT-PCR

Total RNA from the accessory lung lobe was extracted with GRS Total RNA Kit-Tissue (Grisp), according to the manufacturer’s instructions. Spectrophotometer ND-100 UV-visible light (NanoDrop) was used to quantify and assess RNA quality (A260/A280 ≥ 2.0; 1.8 ≤ A260/A230 ≤ 2.2). First-strand cDNA Synthesis Kit (Nzytech) was used to retro-transcribe 1 µg of total RNA per sample. Quantitative PCR was performed on an Applied Biosystems 7500 Fast qPCR system (Applied Biosystems, Thermo Fisher Scientific), using the PowerUp SYBR Green Master Mix (Applied Biosystems, Thermo Fisher Scientific). Data were analysed with 7500 v2.0.6 software (Applied Biosystems, Thermo Fisher Scientific). The following primers were used: *Hif1a*: fwd – GCACTAGACAAAGTTCACCTGAGA, rev – CGCTATCCACATCAAAGCAA; *Glut1*: fwd - CACTGTGGTGTCGCTGTTTG, rev – AAAGATGGCCACGATGCTCA; *Il1b*: fwd - GGGCTGCTTCCAAACCTTTG, rev - AAGACACAGGTAGCTGCCAC; *Vegf*: fwd - AGGGTCAAAAACGAAAGCGC, rev – CGCGAGTCTGTGTTTTTGCA; *Ubb:* fwd – TGGCTATTAATTATTCGGTCTGGAT, rev- GCAAGTGGCTAGAGTGCAGAGTAA. Amplification efficiencies were validated, and expression levels normalized to*Ubb*.

### Immunohistochemistry

Paraffin-embedded lung sections (5 μm thickness) were dewaxed in xylol and rehydrated with ethanol 100%, 90%, 70% and water. For permeabilization, sections were incubated with 0.5% Triton X-100 (Sigma) at room temperature for 10 minutes. For antigen retrieval, sections were heated in a microwave oven (three cycles for 5 minutes each at 800 W) in 0.25 mM citrate buffer (pH 6.0). Endogenous peroxidases were blocked during 20 minutes in methanol containing 0.3% peroxide hydrogen. Unspecific sites were blocked with 5% FBS for 1 hour and the tissue was incubated overnight at room temperature with the primary anti-HIF-1α antibody (H-206) (rabbit polyclonal) 1:25 (Santa Cruz Biotechnology). The secondary antibody was biotinylated IgG (H+L) (Vector Laboratories) 1:250, with further incubation with streptavidin-peroxidase polymer, ultrasensitive (Sigma). After washing, lung sections were developed with 3, 3’-diaminobenzidine (DAB) (Dako Omnis) and counterstained with hematoxylin. Tissues were then dehydrated in an ethanol gradient and mounted with Entellan Mounting Medium (Merck). Slides were analysed on an Olympus BX61 widefield upright microscope.

### Neutrophil viability assay

Bone marrow-derived neutrophils (BMDNs) were isolated by negative selection using MojoSort Mouse Neutrophil Isolation Kit (BioLegend), according to the manufacturer’s instructions. Neutrophil purity was assessed by FACS analysis and was determined to be above 94%. To quantify neutrophil death by lactate dehydrogenase (LDH) release, 2×10^6^ BMDNs were seeded in 24-well plates and incubated for 24 hours with 50 µL of either naked beads or *Af*-beads in a final volume of 2 mL DMEM (Gibco) containing 10% FBS (Gibco), 1% HEPES (Gibco) and 1% penicillin/streptomycin (Wisent). The degree of neutrophil death was determined using CyQUANT LDH cytotoxicity assay (Thermo Fisher) in culture supernatants, following the manufacturer’s instructions.

### Neutrophil antifungal activity

BMDNs were isolated by negative selection using MojoSort Mouse Neutrophil Isolation Kit (BioLegend). Upon isolation, 2×10^5^ BMDNs were seeded in 96 round bottom-well plates in complete DMEM [DMEM (Gibco), 10% FBS (Gibco), 1% penicillin/streptomycin (Sigma), 1% HEPES (Gibco)] containing *A. fumigatus* hyphae (germinated from 3×10^3^ conidia), as previously described (Ralph et al., 2021). Upon 16 hours of co-incubation with hyphae, neutrophils were lysed with sterile water and hyphae were stained for 5 min with 50 µL of a 1 mg/mL Calcofluor white solution (Sigma). Hyphae were washed with sterile water and the staining was quantified using a fluorometer at 360 nm excitation and 440 nm emission.

For the XTT assay, 5×10^5^ neutrophils were suspended in Hank’s balanced salt solution (HBSS) (Gibco) and added to pre-germinated *A. fumigatus* agar beads containing 5×10^4^ conidia (germination was carried out overnight at room temperature followed by 4 hours at 37°C). After 2 hours of co-incubation, neutrophils were lysed with sterile water and incubated 30 min with sterile water at 37°C. Samples were incubated with 100 µL HBSS containing 400 µg/mL XTT (Sigma) and 50 µg/mL coenzyme Q (Sigma) for 2 hours at 37°C. Optical densities were measured at OD 450 nm and 650 nm in a Genesis 20 spectrophotometer (ThermoFisher). The antifungal activity was calculated by the following formula: % antifungal activity=[1-((OD_sample_-OD_neutrophil_)/(OD*_Af_*-OD_blank_))]x100. The OD_sample_ stands for the optical density of neutrophils stimulated with *Af*-beads, OD_neutrophils_, the neutrophils alone and OD*_Af_*, *Af*-beads alone.

### Statistical analysis

Data was analysed with the GraphPad Prism software (version 8.0.1). Normality was assessed for all sets of data and the respective parametric/nonparametric test applied. Data was analysed using Student’s two-tailed t test or Mann-Whitney t test and one-way and two-way analysis of variance (ANOVA) with Bonferroni multiple correction *post-hoc* test, as specified. Statistical significance was defined by p<0.05.

## Results

### HIF-1α is induced during *A. fumigatus* airway infection

**Figure 1:**
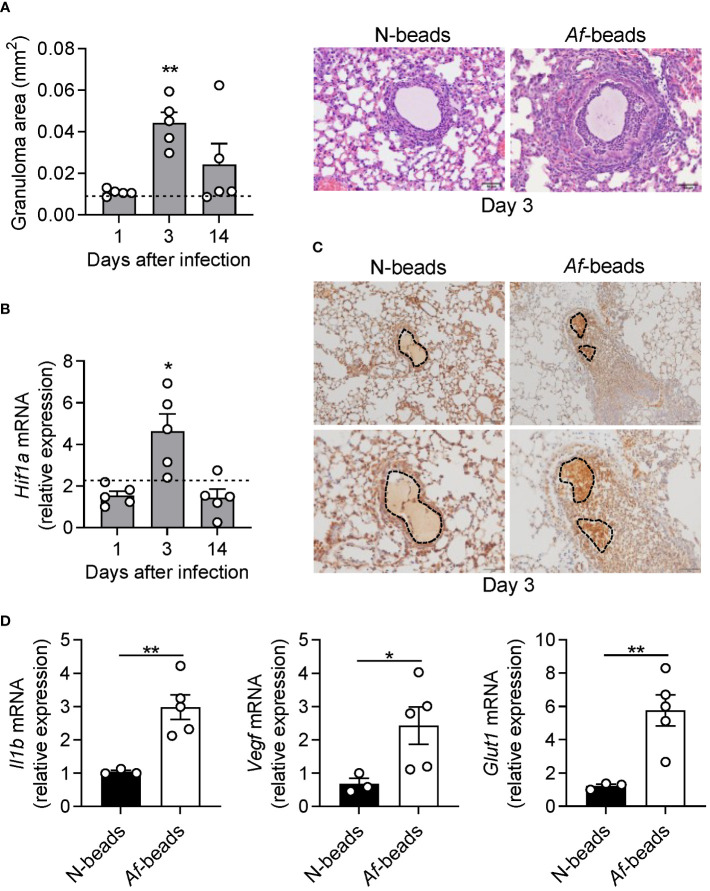
HIF-1α is induced upon *in vivo* development of fungal elicited granuloma-like structures. **(A)** Granuloma area around beads containing *A. fumigatus* conidia (*Af*-beads) after 1, 3 and 14 days post-infection (n = 5). The dotted line represents the mean value of naked bead (N-beads) granulomas after 3 days post-infection (n = 5). Representative hematoxylin and eosin (H&E) staining of mouse lung sections challenged with *Af*-beads or N-beads after 3 days post-infection are shown. Scale bar represents 50 μm. **(B)** Lung mRNA expression of *Hif1a* after 1, 3 and 14 days post-infection with *Af*-beads (n = 5). The dotted line represents the mean value of *Hif1a* expression after 3 days post-infection with N-beads (n = 5). **(C)** Representative immunohistochemistry staining for HIF-1α in lung sections of mice after 3 days post-infection with *Af*-beads or N-beads. Scale bar represents 50 μm. **(D)** Lung mRNA expression of *Il1b*, *Vegf* and *Glut1* after 3 days post-infection with *Af*-beads or N-beads (n = 5). *P < 0.05; **P < 0.01.

Histological analysis of lung sections revealed larger fungal granulomas elicited by beads containing *A. fumigatus* conidia (*Af*-beads) relative to naked (without conidia) bead controls (N-beads) (dotted line) (**Figure 1A**). On day 3 post-infection, when the maximum size was observed, the mean area of the granulomas after *Af*-bead infection was 0.037 mm^2^ and decreased thereafter to 0.026 mm^2^ on day 14 post-infection. Mice challenged with *Af*-beads also displayed increased expression of *Hif1a* in the lungs on day 3 when compared to N-bead controls (dotted line), although the transcript levels decreased on day 14 post-infection (**Figure 1B**), a finding in line with the smaller granuloma areas observed at this timepoint. In support of this, immunohistochemistry analysis on day 3 post-infection also revealed robust HIF-1a staining within granulomatous lesions induced by *Af*-beads compared to the N-beads control (**Figure 1C**). Moreover, the mRNA expression of genes targeted by HIF-1a, including *Il1b*, *Vegf* and *Glut1*, was also increased (**Figure 1D**), suggesting that HIF-1α is induced upon *A. fumigatus* airway infection and fungal-driven granuloma formation.

### Myeloid deletion of HIF-1α restrains granulomatous inflammation and impairs fungal clearance

**Figure 2:**
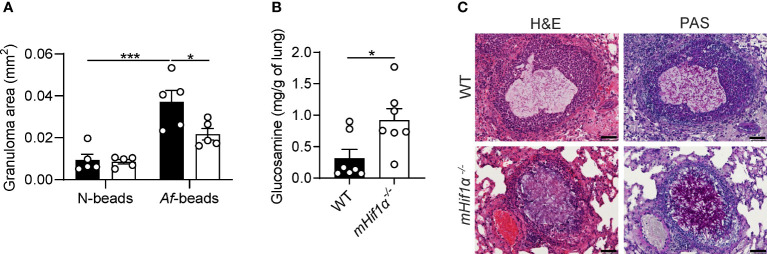
Myeloid deletion of HIF-1α impacts fungal clearance and granuloma establishment. **(A)** Granuloma area around *Af*-beads and N-beads in wild-type (WT) and myeloid HIF-1α-deficient (*mHif1α^-/-^*) mice after 3 days post-infection (n = 5). **(B)** Fungal burden in the lungs of WT and *mHif1α^-/-^* mice after 3 days post-infection with *Af*-beads (n = 7). **(C)** Representative lung sections from WT and *mHif1α^-/-^* mice after 3 days post-infection with *Af*-beads stained with haematoxylin and eosin (H&E) and Periodic acid-Schiff (PAS). Scale bar represents 50 μm. *P < 0.05; ***P < 0.001.

To dissect the function of HIF-1α in fungal granuloma formation, C57BL/6 (wild-type, WT) and HIF-1α myeloid-restricted knock-out *(mHif1α^-/-^*) mice were inoculated with *Af*-beads or N-beads. Our results show that *mHif1α^-/-^* mice developed smaller granulomas when compared to WT mice after challenge with *Af*-beads (**Figure 2A**). No differences were found in the area of the granulomas between WT and *mHif1α^-/-^* mice that were inoculated with N-beads. Although these findings suggest reduced inflammatory pathology, *mHif1α^-/-^* mice instead presented an increased fungal burden in the lungs, as revealed by the glucosamine content (**Figure 2B**), and extensive hyphal growth when compared to WT mice (**Figure 2C**). These results highlight the important role of myeloid HIF-1α in the regulation of inflammation and fungal clearance during fungal granuloma formation.

### *mHif1α^-/-^* mice display impaired neutrophil recruitment

**Figure 3:**
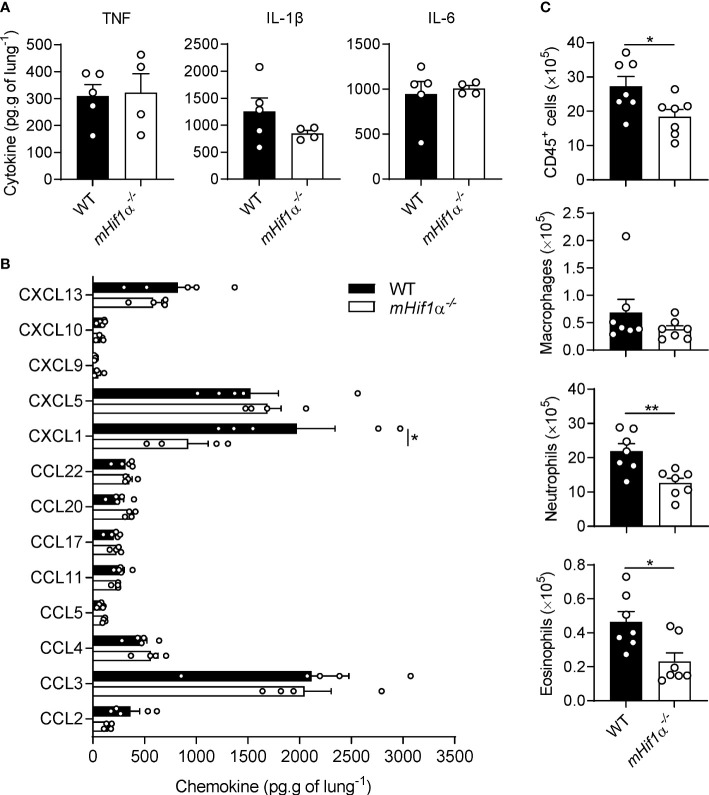
Immune cell recruitment is compromised in *mHif1a* ^-/-^ mice. **(A)** Levels of TNF, IL-1β and IL-6 and **(B)** chemokines in the lungs of WT (n = 5) and *mHif1α^-/-^* (n = 4) mice after 3 days post-infection. **(C)** Immune cell populations in the lungs of WT and *mHif1α^-/-^* mice after 3 days post-infection (n = 7). *P < 0.05; **P < 0.01.

Given the described role of HIF-1α in inflammation and immune chemotaxis, particularly by inducing pro-inflammatory cytokine expression and enhancing the secretion of several chemokines (Krzywinska and Stockmann, 2018), we hypothesized that the smaller area of granulomatous structures found in *mHif1α^-/-^* mice could be owed to one of these factors. Therefore, we quantified several cytokines and chemokines in the lungs of WT and *mHif1α^-/-^* mice in response to *Af*-beads challenge. Although we found no differences in the levels of TNF, IL-6 and IL-1β in lung homogenates between backgrounds (**Figure 3A**), *mHif1α^-/-^* mice presented lower levels of CXCL1, but not other chemokines, than WT mice (**Figure 3B**). Considering that CXCL1 is known to promote neutrophil recruitment (Shepardson et al., 2014), we hypothesized that this finding might also be associated with lower levels of immune cell recruitment. Hence, we evaluated the different immune cell populations of WT and *mHif1α^-/-^* mice challenged with *Af*-beads using flow cytometry. As expected, *mHif1α^-/-^* mice presented decreased levels of total leukocytes (CD45^+^ cells), neutrophils (CD45^+^CD11b^+^Ly6G^+^) and eosinophils (CD45^+^CD11b^+^Ly6G^-^Siglec-F^-^), while no differences were observed in the number of alveolar macrophages (CD45^+^CD11b^-^Ly6G^-^Siglec-F^+^) (**Figure 3C**). Altogether, these results suggest that the impaired granuloma formation in *mHif1α^-/-^* mice may be due to a defective neutrophil recruitment to the lungs.

### Neutrophils lacking HIF-1α display enhanced cell death and defective antifungal mechanisms

**Figure 4:**
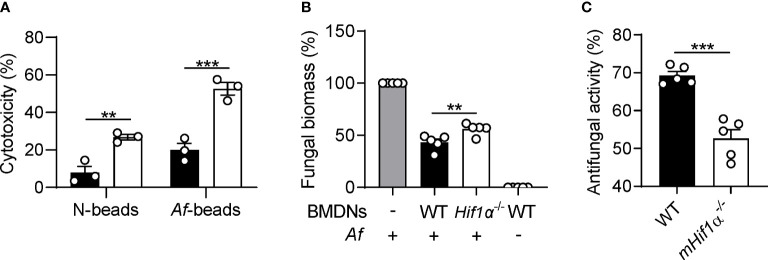
Neutrophils lacking HIF-1α display decreased viability and antifungal activity. **(A)** Neutrophil viability determined by the LDH release assay after 24 hr of co-culture with *Af*-beads or N-beads (n = 3). **(B)** Hyphal growth assessment after 16 hr of co-culture of *A*. *fumigatus* hyphae with BMDNs (n = 5). **(C)** Neutrophil antifungal activity determined by the XTT assay after 2 hr of co-culture with pre-germinated *Af*-beads (n = 5). **P < 0.01; ***P < 0.001.

Because neutrophils are one major cell type involved in the immune response to hyphae (van de Veerdonk et al., 2017) and the granuloma model used involves fungal germination, we decided to further evaluate the role of HIF-1α in neutrophil activity against *Aspergillus* hyphae *in vitro*. To do so, we isolated neutrophils from bone marrow-derived neutrophils (BMDNs) of WT and *mHif1α^-/-^* mice and stimulated them with pre-germinated *Af*-beads and assessed cytotoxicity through the LDH activity assay. Neutrophil viability was significantly reduced in the absence of HIF-1α upon fungal challenge (**Figure 4A**), suggesting an inability of *mHif1α^-/-^* neutrophils to cope with fungal infection. Of note, viability was also lower in *mHif1α^-/-^* neutrophils exposed to N-beads, a finding further supporting a cell-intrinsic defect in viability. Then, we sought to assess whether HIF-1α could also play a role in the antifungal effector mechanisms of neutrophils. Hence, we co-cultured WT and *mHif1α^-/-^* BMDNs with *A. fumigatus* hyphae and evaluated their antifungal activity by assessing hyphal biomass. We observed that neutrophils lacking HIF-1α failed to clear as much hyphae as their WT counterparts, reflected in higher fungal biomass (**Figure 4B**). We also found that *mHif1α^-/-^* BMDNs displayed decreased antifungal activity against pre-germinated *Af*-beads by using the XTT assay (**Figure 4C**). Therefore, our data highlight HIF-1α as a critical driver of neutrophil viability and, consequently, the mechanisms of hyphal clearance, which likely explain the increased fungal burden and extensive hyphal germination detected in conditions of HIF-1α deficiency.

## Discussion

Previous reports established HIF-1α as a central regulator of antifungal immunity in the context of aspergillosis. Specifically, myeloid HIF-1α has been demonstrated to play an essential protective role against invasive pulmonary aspergillosis (Shepardson et al., 2014). This finding suggested that myeloid HIF-1α is required for initiating protective inflammatory signals to control *A. fumigatus* growth and host tissue damage. For example, HIF-1α-mediated regulation of the NLRP3/IL-1β signalling pathway has been reported to play a role in susceptibility to pulmonary aspergillosis in a hyperglycaemia mouse model (Ye et al., 2020). Moreover, HIF-1α stabilization in chronic granulomatous disease (CGD) has been demonstrated to improve infection resolution by promoting LC3-associated phagocytosis (Kyrmizi et al., 2013), a noncanonical autophagy pathway decisive in phagosome maturation and fungal killing (Kyrmizi et al., 2018). However, the general protective role for HIF-1α in response to *Aspergillus* was primarily described in the context of invasive pulmonary disease.

Our results now demonstrate an additional role for HIF-1α in response to a chronic airway stimulation model, in which fungal-elicited granulomatous structures are developed. We demonstrate that HIF-1α deficiency in myeloid cells elicits the formation of smaller granulomas, culminating in uncontrolled fungal growth. We further observe a deficiency in neutrophil recruitment, associated with lower levels of the CXCL1 chemokine. Since the cells that are recruited upon *Af-*beads challenge are mainly neutrophils, these results might help explain the decreased granulomatous inflammation in *mHif1α^-/-^* mice. This observation, along with the defective antifungal clearance, allows an enhanced fungal germination in *mHif1α^-/-^* mice. These results are in line with previous work describing a similar defective mechanism early after an acute model of *A. fumigatus* infection in *mHif1α^-/-^* mice (Shepardson et al., 2014). This indicates that HIF-1α is crucial for the response not only to both invasive and chronic fungal infection but implicates likely overlapping regulatory mechanisms. Importantly, *mHif1α^-/-^* BMDNs have been shown to be fully functional in the phagocytosis and killing of *A. fumigatus* conidia (Shepardson et al., 2014). This suggests that the impaired antifungal clearance observed in our study could be largely attributed to cell-intrinsic defects in neutrophil viability rather than actual defects in effector mechanisms. Importantly, it remains to be assessed what is the role, if any, of eosinophils during the immune response to fungal-elicited granuloma formation.

The model of airway infection used in the present study resorts to encapsulated conidia in agar beads that trigger an immune response upon contact with emerging hyphae or soluble factors produced by hyphae (Urb et al., 2015). In contrast to conidia, *A. fumigatus* hyphae produce the mycotoxin gliotoxin and secrete the polysaccharide galactosaminogalactan, which have been described to induce neutrophil apoptosis (Suen et al., 2001; Robinet et al., 2014). Thus, it is possible that the transcription factor HIF-1α is even of greater importance regarding neutrophil protection from cell death mechanisms in the presence of hyphae and their secreted products. In line with this, our results showed that neutrophils from *mHif1α^-/-^* mice exhibited increased LDH release comparing to wild-type controls upon stimulation with *Af-*beads, suggesting decreased neutrophil viability in conditions of HIF-1α deficiency. In agreement with this, a previous report also showed that *mHif1α^-/-^* neutrophils display increased cell death compared to wild-type cells, following stimulation with the fungal β-glucan derivative curdlan (Shepardson et al., 2014). In the same study, CXCL1 was also described to reduce the apoptosis of *mHif1α^-/-^* neutrophils and affect their recruitment. Likewise, in the model used in our study, CXCL1 responses induced by HIF-1α might not only be required for neutrophil chemotaxis but also be partially involved in controlling neutrophil apoptosis.

In this study, we focused essentially on innate immune cellular responses, as innate immunity is described to be the first line of defence against aspergillosis (van de Veerdonk et al., 2017). At the evaluated time point, adaptive responses are likely not impacted, as *mHif1α^-/-^* mice retain HIF-1α in the lymphoid lineage and chemokines related to lymphocyte recruitment were not altered. However, it is noteworthy that the recruitment of lymphocytes in the lung tissue in this model has been reported to occur with an early peak at 7 days post-infection and a later peak at 28 days-post infection (Urb et al., 2015), thus suggesting that an earlier analysis may not be adequate to evaluate lymphocyte numbers and/or function. Notwithstanding, we did not observe differences in proinflammatory cytokines in *mHif1α^-/-^* mice, even though HIF-1α is described to regulate their expression (Watts and Walmsley, 2019). Of note, this transcription factor is also known to promote cytokine release from dendritic cells in response to *A. fumigatus* (Fliesser et al., 2015), as well as orchestrating the activation of glucose metabolism upon melanin recognition, a crucial mechanism for fungal clearance (Goncalves et al., 2020). Our results do not show differences regarding cytokine production, and a possible explanation might come from a compensatory cytokine production by lung epithelial cells since these are known to respond also to *A. fumigatus* infection and tissue injury with cytokine secretion (de Luca et al., 2010). In line with our findings, Shepardson and colleagues also reported no differences in the cytokine lung content in their model of invasive fungal infection using *mHif1α* ^-/-^ mice (Shepardson et al., 2014).

Collectively, our work provides novel insights into the mechanisms of granulomatous-like inflammation in response to chronic fungal infection. We demonstrate that the transcription factor HIF-1α is crucial to orchestrate a proper immune response in this model and control fungal infection by regulating neutrophil recruitment and survival through the chemokine CXCL1. Henceforth, further studies are needed to fully understand the mechanisms whereby HIF-1α affect disease progression and outcome, posing as a significant progress to overpass current therapeutic limitations and emerging fungal resistance.

## Data availability statement

The original contributions presented in the study are included in the article/Supplementary Material. Further inquiries can be directed to the corresponding author.

## Ethics statement

The animal study was reviewed and approved by Ethics Committee on Life and Health Sciences of the University of Minho (CEICVS).

## Author contributions

SS-F, CC, and AC conceived the study and designed all experiments. SS-F, CD-O, and CB-M developed, performed, and analyzed *in vivo* and *in vitro* models of infection. DA and AM-F performed flow cytometry analysis. ET, SC, and RS analysed data and critically revised the manuscript for important intellectual content. SS-F, CD-O, CC, and AC drafted the manuscript. AC supervised all experiments. All authors contributed to the article and approved the submitted version.

## Funding

This work was supported by the Fundação para a Ciência e a Tecnologia (FCT) (PTDC/MED-OUT/1112/2021, UIDB/50026/2020 and UIDP/50026/2020), the Northern Portugal Regional Operational Programme (NORTE 2020), under the Portugal 2020 Partnership Agreement, through the European Regional Development Fund (ERDF) (NORTE-01-0145-FEDER-000039), the ICVS Scientific Microscopy Platform, member of the national infrastructure PPBI - Portuguese Platform of Bioimaging (PPBI-POCI-01-0145-FEDER-022122), the European Union’s Horizon 2020 research and innovation programme under grant agreement no. 847507, the “la Caixa” Foundation (ID 100010434) and FCT under the agreement LCF/PR/HR17/52190003, and the Gilead Research Scholars Program - Antifungals. Individual support was provided by FCT (SFRH/BD/141127/2018 to CD-O, PD/BD/137680/2018 to DA, SFRH/BD/145955/2019 to CB-M, 2021.07836.BD to AM-F, CEECIND/00185/2020 to RS and CEECIND/04058/2018 to CC).

## Conflict of interest

The authors declare that the research was conducted in the absence of any commercial or financial relationships that could be construed as a potential conflict of interest.

## Publisher’s note

All claims expressed in this article are solely those of the authors and do not necessarily represent those of their affiliated organizations, or those of the publisher, the editors and the reviewers. Any product that may be evaluated in this article, or claim that may be made by its manufacturer, is not guaranteed or endorsed by the publisher.
